# Incidence, mortality and survival trends of penile cancer in Lithuania 1998–2017

**DOI:** 10.3389/fonc.2023.1124101

**Published:** 2023-05-05

**Authors:** Mingaile Drevinskaite, Ausvydas Patasius, Marius Kincius, Justinas Jonušas, Adomas Ladukas, Mindaugas Jievaltas, Laura Kairevice, Giedre Smailyte

**Affiliations:** ^1^Laboratory of Cancer Epidemiology, National Cancer Institute, Vilnius, Lithuania; ^2^Department of Public Health, Institute of Health Sciences, Faculty of Medicine, Vilnius University, Vilnius, Lithuania; ^3^Laboratory of Clinical Oncology, National Cancer Institute, Vilnius, Lithuania; ^4^The Clinic of Internal Diseases, Family Medicine and Oncology, Faculty of Medicine, Vilnius Universitys, Vilnius, Lithuania; ^5^Urology Department, Lithuanian University of Health Sciences, Medicine Academy, Kaunas, Lithuania; ^6^Department of Oncology and Hematology, Institute of Oncology, Medical Faculty, Lithuanian University of Health Science, Kaunas, Lithuania

**Keywords:** penile, cancer, incidence, mortality, survival, epidemiology

## Abstract

**Background and objectives:**

The aim of this study was to analyse trends in penile cancer incidence, mortality, and relative survival in Lithuania during the period of 1998–2017.

**Materials and methods:**

The study was based on all cases of penile cancer reported to the Lithuanian Cancer Registry between 1998 and 2017. Age-specific rates standardized rates were calculated, using the direct method (World standard population). The Joinpoint regression model was used to provide estimated average annual percentage change (AAPC). One-year and five-year relative survival estimates were calculated using period analysis. Relative survival was calculated as the ratio of the observed survival of cancer patients and the expected survival of the underlying general population.

**Results:**

During the study period, the age-standardized incidence rate of penile cancer varied between 0.72 and 1.64 per 100 000, with AAPC 0.9% (95% CI -0.8–2.7). The mortality rate of penile cancer in Lithuania during this period varied from 0.18 to 0.69 per 100 000, with AAPC of -2.6% (95% CI -5.3–0.3). Relative one-year survival of patients, diagnosed with penile cancer improved over the time from 75.84% in period 1998–2001 to 89.33% in period 2014–2017. Relative five-year survival rate of patients, diagnosed with penile cancer changed from 55.44% in period 1998-2001 to 72.90% in period 2014–2017.

**Conclusions:**

The incidence rates of penile cancer showed an increasing trend, while mortality rates were decreasing in Lithuania during 1998-2017. One-year and five-year relative survival increased, however, it does not reach the highest scores of Northern European countries.

## Introduction

Accounting for 1% of all male cancers, penile cancer is a rare malignancy with an incidence of 0.1–1 per 100 000 men in high-income countries (1, 2). According to GLOBOCAN, 36,068 new cases and 13,211 deaths due to the penile cancer were estimated in 2020, respectively. The estimated age-standardized incidence of penile cancer was 0.80 per 100 000 person-years in 2020 worldwide and the incidence is predicted to increase by approximately 56% by 2040 (3). The highest penile cancer incidence rates are still present in developing countries in Southern Africa, South Asia and South America, however, in the most recent analysis significantly increasing age-standardized incidence rates were observed in 13 European countries, one of which is Lithuania (3).

The vast majority of penile cancer cases (95%) are penile squamous cell carcinomas, which arise from the squamous cells of the glanular and preputial skin. Additionally, sarcoma, basal cell carcinoma and melanoma can also occur, but significantly less often (1, 4).

Several risk factors of developing penile cancer have been reported. One of them is infection with human papillomavirus (HPV) types 6, 16, 18, which are associated with penile squamous cell carcinomas (5). Due to the changes in sexual practice and increasing incidence of sexual transmitted diseases and HPV infection, earlier onset of penile cancer diagnosis has been reported (6). Precancerous lesions that are also associated with HPV infection and can increase the risk of invasive penile cancer is erythroplasia of Queyrat and Bowen‘s disease (1, 7). In addition, other risk factors such as tobacco use (8), psoralen UV-A phototherapy to treat psoriasis (9), poor personal hygiene and the presence of a phimosis are also related to penile cancer (10). Phimosis can cause not only accumulation of smegma, which could be carcinogenic as it has been proven in studies with animals, but also retention of microorganisms and lead to infections which are known to be associated with increased risk of penile cancer (11, 12).

Penile cancer can affect men of all ages, however, it is most commonly diagnosed over 60 years of age (13). Less than 50% of patients are diagnosed with localized penile cancer, which has a five-year overall survival of ~90%. Nevertheless, if the cancer spreads, the prognosis worsens dramatically (14). The prognosis of penile cancer is directly related to the lymph node status. Ravi et al. reported the five year survival rate of 95% for patients with negative nodes, 76% with positive inguinal nodes and 0% when the pelvic nodes were metastatic (15). Alongside increasing incidence of penile cancer, some countries have reported increasing mortality rates (7), however, the results from different regions are diverse.

Penile cancer patients are believed to have the longest delay in seeking treatment (waiting time more than 1 year is seen in 15-50% cases) (16). Social stigma is the most common cause for this delay (16). Preventative measures such as awareness of HPV infection and possible oncological consequences for men, which could potentially shorten delays in diagnostics and time to treatment, was lacking. HPV affects the human squamous epithelium in different ways: as a viral infection or viral-associated precancerous lesion when viral genome integrates into the host genome (17). Knowledge of HPV pathogenesis started the development of the vaccine, which has proven to play an important role in female HPV-related cancers‘reduction. However, the results in men population have not been discussed.

From 2023 of February 1, Lithuania is stepping into a new era of gender-neutral HPV vaccination, which will likely change the incidence and mortality trends of penile cancer. Up to this date, there was no published data on penile cancer epidemiological situation in Lithuania. Thus the main purpose of this study was to examine age-specific incidence, mortality trends and relative survival of penile cancer in Lithuania during the period of 1998-2017.

## Material and methods

The study was conducted using the Lithuanian Cancer Registry database covering a population of less than 3 million residents according to 2018 census. Lithuanian Cancer Registry is a population-based cancer registry which contains personal and demographic information (place of residence, sex, date of birth, vital status), information on diagnosis (cancer site, date of diagnosis, method of cancer verification) and death (date of death, cause of death) of all cancer patients in Lithuania since 1978. Cancer registry data is included in Cancer Incidence in Five Continents, where submitted data undergo systematic evaluation of indices of completeness and accuracy (18). The study included all cases of cancer of the penis reported to the Registry during 1998–2017 (ICD-10 codes C60.0–C60.9 for penile cancer). Stage of the disease was based on TNM classification. Penile cancers (21 cases) notified only by the death certificate (DCO) were excluded from survival analysis.

Age-specific and age-standardized incidence rates were calculated. Standardization was performed using the direct method (World standard population). Corresponding population data, by age, sex and year were available from Statistics Lithuania. Age-standardized rates were calculated each year. The Joinpoint regression model was used to provide estimated average annual percentage change (AAPC) and with 0 number of joinpoint allowed, to detect estimated trend over the analysed period. For each of the identified trends, we also fit a regression line to the natural logarithm of the rates using calendar year as a regression variable. 95% confidence intervals for AAPC were calculated as well. Changes were considered statistically significant if p<0.05. The Joinpoint software version 4.3.1.0 was used.

One-year and five-year relative survival estimates were calculated using period analysis. The relative survival was calculated as the ratio of the observed survival of cancer patients and the expected survival of the underlying general population, and it was calculated according to the Ederer II method using national life tables for Lithuanian population stratified by age, gender and calendar year. All calculations were conducted with STATA 15 (StataCorp LP, College Station, TC, USA); relative survival analysis was performed with the strs module.

## Results

**Figure 1:**
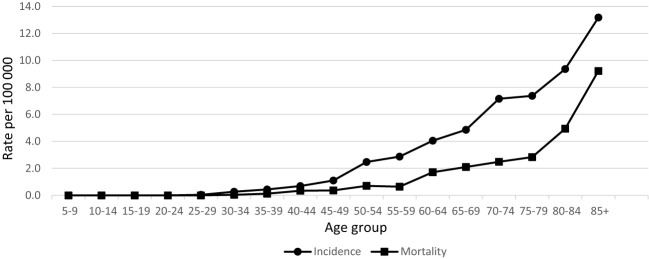
Age-specific incidence and mortality rates of penile cancer in Lithuania in 1998–2017.

**Figure 2:**
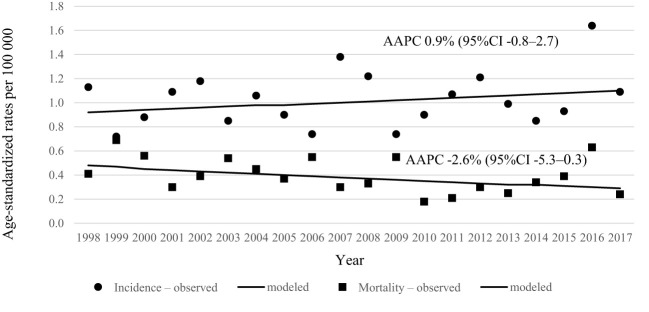
Age-standardized incidence and mortality trends of penile cancer in Lithuania in 1998–2017.

During 20 years’ period in Lithuania, 464 penile cancer cases were diagnosed. Data quality was consistent over the years as per morphological verification and only few diagnoses were registered as death certificated only cases (**Table 1**). Incidence and mortality rates of penile cancer increased with age (**Figure 1**). Age-standardized incidence of penile cancer varied between 0.72 and 1.64 per 100 000 during study period. The age-standardized incidence rate increased insignificantly with AAPC 0.9% (95% CI -0.8–2.7). The mortality rate of penile cancer in Lithuania during this period varied between 0.18 and 0.69 per 100 000. The mortality rate of penile cancer insignificantly decreased, with AAPC of -2.6% (95% CI -5.3–0.3) (**Figure 2**).

**Table 1:** Data quality indicators of cancer registry data on penile cancer in Lithuania in 1998-2017.

Years	Cases	Morphological verification (%)	Death certificate only (%)
1998	23	21 (91.30%)	0 (0.00%)
1999	15	15 (100.00%)	0 (0.00%)
2000	20	19 (95.00%)	0 (0.00%)
2001	23	22 (95.65%)	0 (0.00%)
2002	26	24 (92.30%)	0 (0.00%)
2003	18	15 (83.33%)	0 (0.00%)
2004	23	23 (100.00%)	0 (0.00%)
2005	21	19 (90.47%)	0 (0.00%)
2006	16	13 (81.25%)	0 (0.00%)
2007	31	25 (80.64%)	3 (9.68%)
2008	28	28 (100.00%)	0 (0.00%)
2009	17	16 (94.11%)	0 (0.00%)
2010	21	19 (90.47%)	0 (0.00%)
2011	25	24 (96.00%)	0 (0.00%)
2012	30	27 (90.00%)	1 (3.33%)
2013	22	21 (95.45%)	1 (4.55%)
2014	18	16 (88.89%)	2 (11.11%)
2015	22	20 (90.90%)	2 (9.10%)
2016	38	31 (81.58%)	6 (15.79%)
2017	27	25 (92.59%)	2 (7.41%)

**Figure 3:**
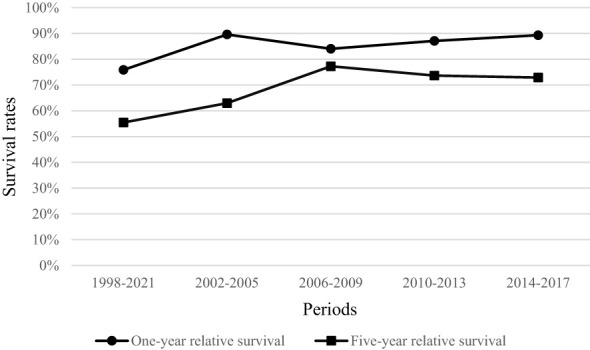
Relative one-year and five-year survival of penile cancer patients in Lithuania by period of diagnosis.

Relative to the general population, one-year survival rate of patients, diagnosed with penile cancer was 85.40% (95% CI 81.24–88.90). Stage-specific relative survival ranged from 101.36 for patients with stage I to 28.88% with stage IV penile cancer (**Table 2**). Relative five-year survival rate of patients, diagnosed with penile cancer was 68.92% (95% CI 62.73–74.80). Stage-specific relative survival ranged from 97.51% (95% CI 86.74–106.04) for patients with stage I penile cancer to 3.0% (95% CI 0.02–13.52) with stage IV (**Table 3**). Relative one-year survival of patients, diagnosed with penile cancer improved over the time from 75.84% in period 1998–2001 to 89.33% in period 2014–2017. Relative five-year survival rate of patients, diagnosed with penile cancer changed from 55.44% in period 1998–2001 to 72.90% in period 2014–2017 (**Figure 3**).

**Table 2:** One-year relative survival of patients with penile cancer in Lithuania in 1998–2017.

Stage	Cases	Deaths	Relative survival, % (95 % CI)	
Total	464	83	85.40	81.24–88.90
Stage I	138	5	101.36	96.25–103.57
Stage II	179	28	88.30	81.81–93.08
Stage III	71	17	80.35	67.98–89.14
Stage IV	40	29	28.88	15.61–43.82
Not reported	36	4	78.20	46.52–94.96

**Table 3:** Five-year relative survival of patients with penile cancer in Lithuania in 1998-2017.

Stage	Cases	Deaths	Relative survival, % (95 % CI)	
Total	464	207	68.92	62.73–74.80
Stage I	138	35	97.51	86.74–106.04
Stage II	179	83	68.65	58.89–77.64
Stage III	71	34	52.86	38.06–67.23
Stage IV	40	39	3.00	0.02–13.52
Not reported	36	8	61.10	27.79–90.00

## Discussion

We present the first study to examine penile cancer’s incidence, mortality and relative survival trends in Lithuania during the period 1998–2017. Incidence rates were increasing during study period, while mortality decreased; increase of relative survival rates also was observed.

Globally, the estimated age-standartized incident was 0.8 per 100 000, according to GLOBOCAN (3). The highest incidence rates of penile cancer were reported in Uganda (2.2 per 100 000) and Brazil (2.1 per 100 000) (3). Israel reports the lowest incidence rates of penile cancer close to zero which has been attributed to high neonatal circumcision prevalence (19). In our study we observed similar incidence rates compared to other European countries. Incidence rate of penile cancer in Norway was 0.91 per 100 000 for the period of 2011–2015 and in Denmark 1.3 per 100 000 from 2006 to 2008 (7, 20). In our study age-standardized incidence varied between 0.72 and 1.64 per 100 000 during study period. The incidence of penile cancer is increasing in many countries worldwide (1, 3). Out of 23 studied European regions, only two countries reported a decrease in penile cancer’s incidence – Switzerland and France (3).

There are a number of reasons for the increasing incidence trends of penile cancer worldwide, one could be decreasing rates of circumcision. Nowadays, worldwide circumcision rate is low at 38% with the highest rates in countries where circumcision is part of the religion and culture (11, 21). Circumcision not only helps to reduce sexually transmitted infections, including HPV and human immunodeficiency virus, but also reduces infections such as balanoposthitis which can all cause penile cancer (22). HPV infection remains the major risk factor for penile cancer. Persistent exposure to HPV leads to the integration of HPV DNA into the host genome and eventual cell transformation into a malignant phenotype (23). Changes in unprotected sexual behaviour worldwide can partly explain an increasing number of male patients developing penile cancer before 60 years of age as it is discussed in Norwegian retrospective study (7).

According to EUROCARE-5, estimated one-year relative survival of penile cancer patients was 86% and five-year relative survival was 68% in Europe overall. The highest five-year relative survival was observed in Norway (83%) and Denmark (82%) and the lowest in Slovakia (50%). EUROCARE-5 reports that the one-year relative survival of penile cancer patients in Lithuania was 85.5% and five-year relative survival was 60.6% (period of diagnosis from 1999 to 2007) (24). In our study, we observed rather the same results as it is stated in EUROCARE-5: general one-year relative survival for all stages was 85.4% and five-year relative survival was 68.9% for patients diagnosed during the period 1998-2017. However, we observed a relative survival’s improvement during the different periods of time: one-year relative survival improved from 75.84% in period 1998-2001 to 89.33% during the period of 2014 and 2017, and five-year relative survival improved from 55.44% in period 1998-2001 to 72.90% in period 2014-2017. Relative survival of penile cancer patients has also shown improvement in some Nordic countries: five-year relative survival increased in Norway and in Denmark. In Norway, during the period of 1984-1988, five-year relative survival was 69% and in 1999-2003 it increased to 80% (7).

Globally, the estimated age-standardized mortality rate of penile cancer was 0.29 per 100 000 in 2020 (3). Higher mortality rates were observed nearly twice as high in low- and middle-income countries as in high-income countries. Age-standardized mortality trends of penile cancer among men in our study varied between 0.18 and 0.69 per 100 000. In a mentioned before study, Norwegians have observed increased mortality trend over the study period and in United States of America incidence-based mortality showed an initial significant increase from 2000 to 2002 followed by a deceleration rate during 2002 to 2018 (7, 25).

Penile cancer can potentially have significant social concerns for those who affected. There is a certain level of stigma and shame associated with penile cancer due to its association with sexual behaviour. This can lead to feelings of embarrassment, isolation, and reluctance to seek medical care (16). Moreover, penile cancer can have impact on sexual functioning, including erectile dysfunction, which can affect the quality of life and relationships (26). All of the mentioned above fears can delay diagnostics and the time to initiate treatment. We observed a decreasing penile cancer mortality trend and improved relative survival, which could suggest positive changes in diagnostics, treatment and patients’ awareness of symptoms during the studied period. We believe that more and more clinicians are using approved guidelines to treat the patients and implementation of adequate surgical techniques as dynamic sentinel node biopsy and modified lymphadenectomy played a crucial part in improved survival results (27). However, our stated results do not reach the highest reported survival results of Northern Europe.

There are a number of potential reasons why penile cancer survival in Lithuania is unsatisfactory. Penile cancer is a rare malignancy which comprises only <1% of all cancer worldwide. In Lithuania, due to the rarity of this disease, we get only ~22 patients per year, who are treated in different medical centers where different diagnostic and treatment guidelines are approached by urologists, radiotherapists and oncologists, with most of them following European Association of Urology, European Society for Medical Oncology and National Comprehensive Cancer Network guidelines for penile cancer. The wide consensus among rare cancer experts is explained by Sandrucci et al. that in order to improve patients’ outcomes, patients with rare cancers should be treated at centers from the beginning of the disease with a multidisciplinary clinical approach (28).

In addition to improving survival, more attention should be paid on prevention. The World Health Organization considers vaccination to be one of the key elements of primary healthcare and universal healthcare coverage (29). As at December 2021, all European Union countries have introduced HPV vaccination in their national programmes (30). Previously, those vaccines have been exclusively available for women from 9 to 26 years of age only. However, gender-neutral vaccination has now been adopted in majority of European countries to prevent cervical, vulvar, oropharyngeal, anal and penile cancer (31). Regarding male HPV cases, there was a concern in the low rate of seroconversion after natural infection, however, the quadrivalent HPV vaccine has been proven to be highly immunogenic in men age 16 to 26 (32, 33). Taking into consideration that HPV is found in 42% of penile carcinomas and 90% of dysplasias (34), it is believed that universal HPV vaccination is likely to be more effective and efficient in reducing HPV virus circulation and cancer related to it in the general population even at lower levels of vaccine uptake (35). One randomized, placebo-controlled, double-blind trial of 4065 healthy 16-26 years old males, showed that quadrivalent vaccine reduced the incidence of lesions related to HPV6, HPV11, HPV16 and HPV18 by 90% compared with those patients who received a placebo (32). However, the vaccination numbers globally are low, in 2019 only about 4% of boys had received the full course of the vaccine compared to 15% of girls (36). Just a few countries have reached 70% vaccination coverage for girls. Taking into account lower-middle-income countries, HPV vaccination coverage is 16% for the first dose and 12% for the second dose (36). The most recent analysis on gender-neutral HPV vaccination emphasised the financial burden for lower income countries, the new prospects of single dose vaccine and gender equality in order to expand cancer prevention globally (37). According to the National Public Health Centre under the Ministry of Health of Lithuania statistics, 64,4% of 11 years old girls were vaccinated the first dose of HPV vaccine and in 2020 the vaccination numbers decreased by ~3% (38). Although it is recommended to vaccine boys in Lithuania, unfortunately, there is no reliable statistics about the vast of the vaccination. However, from this year February Ist, Lithuania is adapting gender-neutral HPV vaccination on the national routine immunization schedule.

Our study is the first study in Lithuania to examine penile cancer incidence, mortality and relative survival. However, several limitations should be pointed out. Firstly, the reliability of our results depends on the quality of cancer registration. Changes in completeness of registration over time may have artificially influenced temporal trends to some extent. However the fact that with increasing incidence we observed increasing survival and decreasing mortality supports a non-artifactual effect. Secondly, the lack of statistical information upon prevalence of HPV infection’s epidemiological situation and vaccination among boys in Lithuania. Future studies should be done to explore HPV infection related penile cancer trends and the impact on gender-neutral vaccination.

## Conclusions

The incidence rates of penile cancer showed an increasing trend, while mortality rates were decreasing in Lithuania during 1998-2017. One-year and five-year relative survival increased, however, it does not reach the highest scores of Northern European countries. To improve our patients’ outcomes, more attention should be paid to preventative measures such as gender-neutral vaccinations, early diagnosis and multidisciplinary approach for diagnostics and management in dedicated centres, involving precise staging, adequate treatment, and attentive follow-up.

## Data availability statement

The raw data supporting the conclusions of this article will be made available by the authors, without undue reservation.

## Ethics statement

Ethical review and approval was not required for the study on human participants in accordance with the local legislation and institutional requirements. Written informed consent for participation was not required for this study in accordance with the national legislation and the institutional requirements. Written informed consent was not obtained from the individual(s) for the publication of any potentially identifiable images or data included in this article.

## Author contributions

Conceptualization: MD, JJ and GS. Data curation: MJ and GS. Formal analysis: AP. Investigation: GS. Methodology: AP and GS. Project administration: MJ. Resources: MJ. Supervision: GS. Validation: MK and GS. Visualization: MD, AP, MK, JJ, AL, LK and GS. Writing – original draft: MD. Writing – review & editing: AP, MK, JJ, AL, MJ, LK and GS. All authors contributed to the article and approved the submitted version.
